# Clinical evaluation of two pathogen enrichment approaches for next-generation sequencing in the diagnosis of lower respiratory tract infections

**DOI:** 10.1128/spectrum.00922-25

**Published:** 2025-06-11

**Authors:** Xiao Lei, Xiumei Xu, Chao Liu, Lipeng Zhong, Shupeng Yin, Biaoxian Li, Ling Cao, Zhiting Xie, Jing Li, Xuan Zhang, Yaping Guo, Liang Zhang, Haiyan Lin, Sufeng Zhang, Chengsheng Zhang, Tian Gong

**Affiliations:** 1 Center for Molecular Diagnosis and Precision Medicine, The First Affiliated Hospital, Jiangxi Medical College, Nanchang University 117970, Nanchang, China; 2 Department of Medical Genetics, The First Affiliated Hospital, Jiangxi Medical College, Nanchang University 117970, Nanchang, China; 3 Jiangxi Provincial Center for Advanced Diagnostic Technology and Precision Medicine, The First Affiliated Hospital, Jiangxi Medical College, Nanchang University 117970, Nanchang, China; 4 School of Public Health, Jiangxi Medical College, Nanchang University 568737, Nanchang, China; 5 Medical Department, Hangzhou Pan-omics Biotechnology Co., Ltd., Hangzhou, China; Children's National Hospital, George Washington University, Washington, DC, USA

**Keywords:** lower respiratory tract infection, targeted next-generation sequencing, multiplex PCR, pathogen enrichment, etiological diagnosis

## Abstract

The underdevelopment of microbiological tests has contributed to diagnostic delay and inappropriate use of antibiotics in patients with lower respiratory tract infections, which is ranked as the seventh leading cause of death globally. Next-generation sequencing (NGS) has emerged as a promising platform for the diagnosis of infectious diseases, albeit with high costs and challenges in result interpretation. Here we evaluated two NGS-based pathogen detection assays for the etiological diagnosis of pneumonia in a prospective cohort of 257 patients. Both assays utilized multiplex polymerase chain reaction (PCR) for pathogen enrichment. One assay was designed to promiscuously amplify and identify more than 1,000 pathogens (broad-spectrum targeted next-generation sequencing [bs-tNGS]), while the other specifically targeted 194 pathogens (pathogen-specific targeted next-generation sequencing [ps-tNGS]). The analytical and diagnostic performances of both assays were compared using a composite clinical reference standard. The specificity of ps-tNGS was higher than that of bs-tNGS (84.85% vs. 75.00%), while the sensitivities of both assays were similar (>89%). In addition, a significant overlap in the frequently detected pathogens by the two methods was observed. Moreover, the enrichment of pathogens via multiplex PCR for ps-tNGS has alleviated the requirement for deep sequencing in the shotgun metagenomic workflows and thus dramatically lowered the assay cost. This study demonstrated that ps-tNGS achieved a better overall diagnostic performance and may potentially replace bs-tNGS in the clinical application.

**IMPORTANCE:**

Microbial enrichment in metagenomic next-generation sequencing has been achieved through differential cell lysis, but the results varied, depending on experimental procedures and sample types. Therefore, direct enrichment of pathogen DNA/RNA was attempted via multiplex PCR or hybrid probe capture (targeted next-generation sequencing [tNGS]). We evaluated two enrichment methods based on multiplex PCR. One method utilized a primer design strategy to amplify over 1,000 respiratory pathogens (bs-tNGS), while the other specifically targeted 194 pathogens (ps-tNGS). Our findings disavowed the notion that “the more, the better” in tNGS workflows, since ps-tNGS exhibited equivalent sensitivity and, notably, higher specificity than bs-tNGS in a prospective cohort of 257 patients who were suspected of having pneumonia. In future evaluations of tNGS assays, researchers should pay more attention to diagnostic specificity, rather than focusing solely on sensitivity, since a low specificity may potentially lead to misdiagnosis and overuse of antibiotics in cases of non-infectious diseases.

## INTRODUCTION

The Global Burden of Diseases, Injuries, and Risk Factors Study estimated that lower respiratory tract infections (LRTIs), including pneumonia, bronchitis, and bronchiolitis, were ranked as the seventh leading cause of death in 2021 (1). Identification of the causative pathogens is challenging due to limitations of conventional microbiological tests (CMTs), and the etiology for approximately 40% of patients with community-acquired pneumonia (CAP) was undetermined (2). In another study, no pathogens were detected in 41.7% of adult patients with acute cough and suspected LRTIs (3). Without timely identification of pathogens, effective treatment strategies could not be determined and thus may lead to treatment delay and improper use of antibiotics.

CMTs for diagnosing LRTIs include the measurement of inflammatory biomarkers (such as C-reactive protein and procalcitonin), bacterial and fungal cultures of respiratory specimens and blood, matrix-assisted laser desorption/ionization time-of-flight mass spectrometry, serological tests, and nucleic acid amplification tests, such as polymerase chain reaction (PCR) (4–7). CMTs often rely on successful culture of pathogens, which limits detection of fastidious or unculturable organisms and delays diagnosis by 1–5 days (7–10). Moreover, PCR assays may suffer from mismatches of primers with mutant pathogens, such as the severe acute respiratory syndrome coronavirus 2 (SARS-CoV-2) variants of concern with mutations escaping diagnostic PCR (11).

To further expand the detection spectrum, shotgun metagenomic next-generation sequencing (mNGS) was introduced in 2014 for the clinical diagnosis of leptospirosis from cerebrospinal fluid (12) and later applied to other infectious diseases and patient specimens (13). Being culture-independent and hypothesis-free, mNGS has been shown to be more sensitive than CMTs and can theoretically identify all microorganisms of known genomic sequences (14). However, the unbiased nature of mNGS and overabundance of host nucleic acids in patient specimens have led to the requirement of deep sequencing depth (around 20 million reads) in order to achieve a satisfying diagnostic performance (15). Host-depletion methods were thus developed to reduce the host background prior to library preparation, but the results varied among studies (16, 17). As a result, direct enrichment of microorganisms was attempted through multiplex PCR (18) or hybrid capture (19), both of which can significantly reduce the interference of host DNA and enhance assay sensitivities.

In this study, we compared the analytical and diagnostic performances of two next-generation sequencing (NGS) assays that took advantage of pathogen enrichment by multiplex PCR in a cohort of 257 inpatients who were preliminarily diagnosed with LRTIs. One assay utilized primers to amplify both species-specific as well as housekeeping marker genes/regions that were conserved among bacterial and fungal species (see Materials and Methods) in order to identify a broad spectrum of pathogens (broad-spectrum targeted next-generation sequencing [bs-tNGS]). The other assay included only species-specific primers to amplify 194 respiratory pathogens (pathogen-specific targeted next-generation sequencing [ps-tNGS]), which was a standardized enrichment strategy used in amplicon sequencing workflows (20). The respiratory specimens were obtained from each patient and tested simultaneously by CMTs and both targeted next-generation sequencing (tNGS) assays.

## MATERIALS AND METHODS

### Patient recruitment and sample collection

A total of 257 respiratory tract samples were collected from 257 inpatients with LRTIs at The First Affiliated Hospital of Nanchang University. The patient selection criteria include initial diagnosis of pneumonia (positive radiographic findings by chest X-ray or CT and clinical presentations, including cough, sputum production, fever, and shortness of breath). Patients with incomplete or missing medical documentation, those contraindicated for bronchoscopy, or cases with insufficient bronchoalveolar lavage fluid (BALF) samples (<10 mL) were excluded from this study. These samples consisted of 247 BALFs, 4 lung biopsy samples, 3 sputum samples, and 3 pleural effusions. Total nucleic acid (DNA and RNA) was extracted from either 1 mL of each respiratory liquid sample or a 3 mm × 3 mm ×3 mm tissue. Reverse transcription-PCR was then performed to generate complementary DNA (cDNA) from the RNA (Hangzhou MatriDx Biotechnology, #MD005; Hangzhou Allsheng Instrument, Autopure 20B). The resulting DNA and cDNA mixture was then divided into two aliquots that underwent bs-tNGS and ps-tNGS experimental procedures, respectively.

### Conventional microbiological tests

The CMTs included smear, acid-fast stain, bacterial/fungal culture, 1,3-β-d-glucan test, galactomannan antigen test, T-SPOT.TB test, as well as nucleic acid amplification tests for *Mycobacterium tuberculosis*, Epstein-Barr virus (EBV), and cytomegalovirus (CMV). The CMTs were requested at the clinician’s discretion, and in several cases, only a subset of these tests was conducted.

### Targeted next-generation sequencing

An internal control DNA that shared no sequence homology with known pathogens was synthesized, amplified by PCR (TAKARA PrimeSTAR HS DNA Polymerase, Cat #R044), purified using magnetic beads (MatriDx, Cat #MD012), and added to the BALF sample prior to nucleic acid extraction at a concentration of 0.02 ng/µL. The PCR program for DNA pathogens was 1 cycle at 95°C for 30 s, 35 cycles at 95°C for 30 s, 60°C for 90 s, and 72°C for 30 s. For RNA viruses, the PCR program was 1 cycle at 50°C for 10 min (reverse transcription), 1 cycle at 95°C for 2.5 min, 40 cycles at 94°C for 15 s, and 58°C for 30 s. The amplicons from both reactions were mixed and underwent PCR-free library preparation according to a previous protocol (15). Library concentrations were quantified by real-time PCR (MatriDx, #MD057) and pooled. Single-end 50 bp sequencing was carried out on Illumina NextSeq, and approximately one million clean reads (minimum 0.1 M) were obtained for each library. For each run, two negative controls (cell culture medium containing 10^4^/mL JURKAT cells and nuclease-free water) and one positive control (*Klebsiella pneumoniae* at 5 × 10^2^ cp/mL, Epstein-Barr virus at 1 × 10^3^ cp/mL, *Candida albicans* at 1 × 10^4^ cp/mL, and influenza A virus at 1 × 10^3^ cp/mL were mixed with 10^5^/mL JURKAT cells) were included for quality control. Raw sequences were processed by a bioinformatic pipeline: (i) adapter sequences and low-quality bases (*Q*-score cutoff of 20) were trimmed; (ii) host reads were filtered by mapping to the human reference genome (GRCh38.p13) using Burrows-Wheeler alignment (BWA, http://bio-bwa.sourceforge.net); and (iii) after removal of low-complexity reads, the remaining sequences were aligned by BWA to an in-house reference database (curated from the National Center for Biotechnology Information Nucleotide database, GenBank, and whole-genome sequencing data) in order to identify microbial species. Unique microbial reads were reported if (i) the sequencing data passed quality control filters (library concentration >1 pM, Q20 >85%, Q30 >80%); (ii) negative control in the same sequencing run does not contain the species or the reads per million (RPM) of the sample/RPM of the negative controls ≥5; and (iii) all species spiked in the positive control were detected.

### Primer design rationale for pathogen enrichment

The PCR primers in the bs-tNGS assay were designed in order to enrich and identify more than 1,000 pathogen species, which was achieved by including species-specific sequences (for DNA and RNA viruses) as well as housekeeping marker genes/regions, including 16S rRNA (21), topA, rpoB, and valS, as reported previously (22) for bacteria and internal transcribed spacer (ITS) (23) for fungi. Similar to a previous study (22), the marker genes/regions were selected according to the following criteria: (i) the targeted sequence was conserved and present in all or most species; (ii) the nucleotide composition of the amplicons was variable enough to provide species/strain-level resolution; (iii) the amplicons were flanked by highly conserved sequences to minimize the requirement of degenerate primers to cover all possible nucleotide permutations for any given target. In contrast, for the ps-tNGS assay, the primers were designed in a species-specific manner, with several clinically important pathogens having more than one pair of primers to increase detection sensitivity (such as *M. tuberculosis*, *Aspergillus*, and cryptococcal species). The rationale for the 194-pathogen panel was based on previous studies including mNGS in pneumonia patients as well as the Community-Acquired Pneumonia (CAP)-China publications, from which we chose the most common respiratory pathogens and less common but highly virulent pathogens (15, 24–26).

## RESULTS

### Positivity and concordance

**Fig 1:**
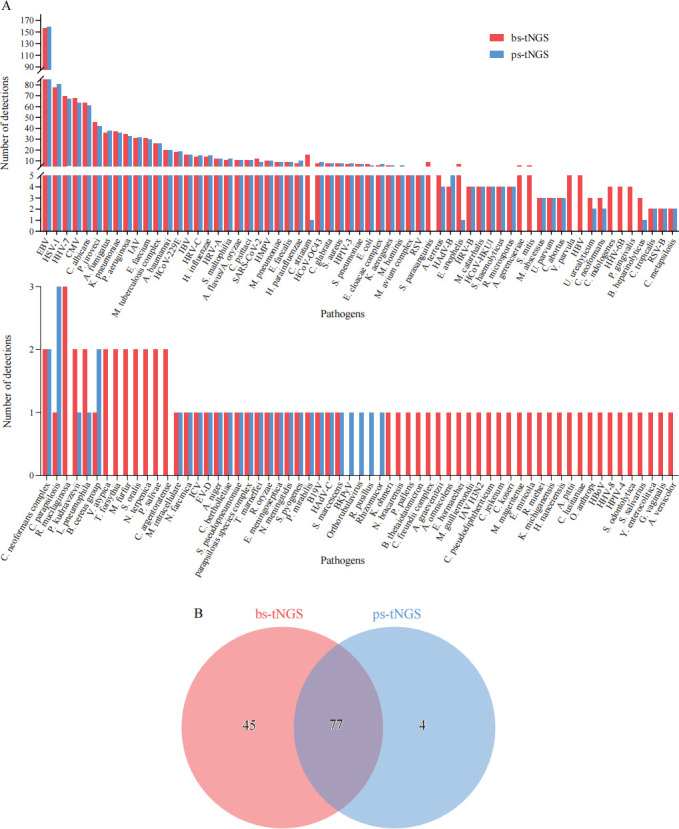
Frequency distribution of detected pathogens by bs-tNGS and ps-tNGS. (**A**) Colored bars indicated the detection rate for pathogens reported by two assays. Sorted from high to low, the 1st–63rd pathogens are graphed on top, while the 64th–126th pathogens are shown below. (**B**) Venn diagram showing the number of microbial species detected by both methods.

The clinical characteristics of enrolled patients are summarized in Table 1. The positive rate was 97.3% (250 out of 257) and 97.7% (25 out of 257) for the bs-tNGS and ps-tNGS assays, respectively. A total of 122 and 81 microbial species were reported by the bs-tNGS and ps-tNGS assays, respectively (Fig. 1A; Table S1). Detected pathogens were fully concordant (same or both negative) in 51.0% (131 out of 257), partial match (at least one pathogen matched) in 46.7% (120 out of 257), and no match in 2.3% (6 out of 257) of specimens (Table 2). The no-match samples were due to the inability of ps-tNGS to detect several microorganisms covered by bs-tNGS, 10 of which were of low pathogenicity and beyond the detection scope of ps-tNGS. Moreover, bs-tNGS missed four pathogens (BKPyV, *Orthorubulavirus*, *Rhizomucor pusillus*, and *Rhizomucor*) when compared to ps-tNGS, which on the other hand, missed 35 potential pathogens that are known to cause LRTIs (Fig. 1B).

**TABLE 1:** Clinical characteristics of the enrolled patients^*a*^

No. of patients	257
Male	171 (66.53)
Age (years)	59.07 ± 18.03
Clinical symptoms	
Cough	189 (73.54)
Expectoration	152 (59.14)
Fever	125 (48.64)
Chest tightness	98 (38.13)
Shortness of breath	98 (38.13)
Hemoptysis	30 (11.67)
Chest pain	17 (6.61)
Underlying conditions, *n* (%)	
Cancer	57 (22.18)
Chronic obstructive pulmonary disease	45 (17.44)
Hypertension	44 (17.12)
Diabetes mellitus	35 (13.62)
Cardiovascular and cerebrovascular disease	27 (10.51)
Hepatitis	17 (6.61)
Bronchiectasis	15 (5.84)
Chronic kidney disease	12 (4.70)
AIDS	3 (1.16)
Asthma	3 (1.16%)
Routine laboratory tests	
WBC count (10^9^/L)	7.03 (5.15–12.19)
Neutrophil count (10^9^/L)	5.14 (3.13–10.54)
Lymphocyte count (10^9^/L)	1.01 (0.59–1.49)
CRP (mg/L)	43.28 (10.27–115.23)
PCT (ng/mL)	1.62 (0.35–6.21)

^*a*^ AIDS, acquired immunodeficiency syndrome; CRP, C-reactive protein; PCT, procalcitonin; WBC, white blood cell. Some variables are shown as the number (percentage, %) while others are shown as median (quartile Q1–Q3).

**TABLE 2:** Comparison between bs-tNGS and ps-tNGS^*a*^

	bs-tNGS	ps-tNGS
Positive rate (%)	97.3	97.7
Turnaround time (h)	13	13
Cost	More expensive	60% cheaper than bs-tNGS
Sequencing depth	1 million reads	1 million reads
Results fully match (%)	51.0	
Results partial match (%)	46.7	
Results no match (%)	2.3	
Sensitivity	89.81% (84.99%–93.51%)	89.95% (85.19%–93.60%)
Specificity	75.00% (57.80%–87.88%)	84.85% (68.10%–94.89%)
PPV	95.57% (92.42%–97.44%)	97.52% (94.61%–98.88%)
NPV	55.10% (44.18%–65.55%)	56.00% (45.50%–65.99%)
Accuracy	87.70% (83.00%–91.49%)	89.29% (84.79%–92.82%)

^*a*^ NPV, negative predictive value; PPV, positive predictive value. The minimum sequencing depth for both assays was 0.1 million reads. The numbers shown in parentheses indicate 95% confidence intervals.

### Pathogen prevalence

**Fig 2:**
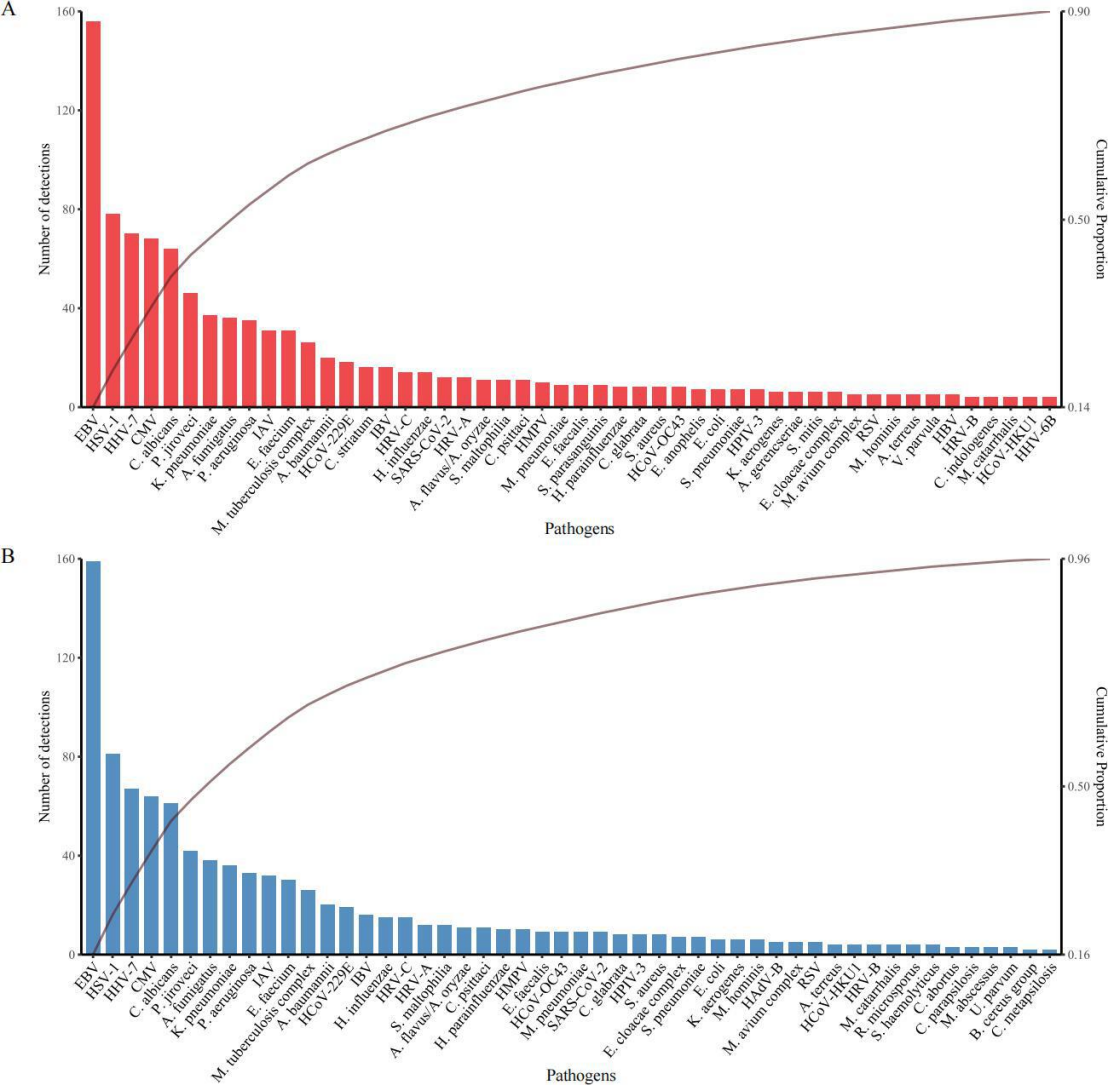
Frequency distribution of reported pathogens by bs-tNGS and ps-tNGS. (**A**) The top 50 most commonly reported pathogens and the corresponding number of detections are shown for bs-tNGS. (**B**) The top 50 most commonly reported pathogens and the corresponding number of detections are shown for ps-tNGS.

**Fig 3:**
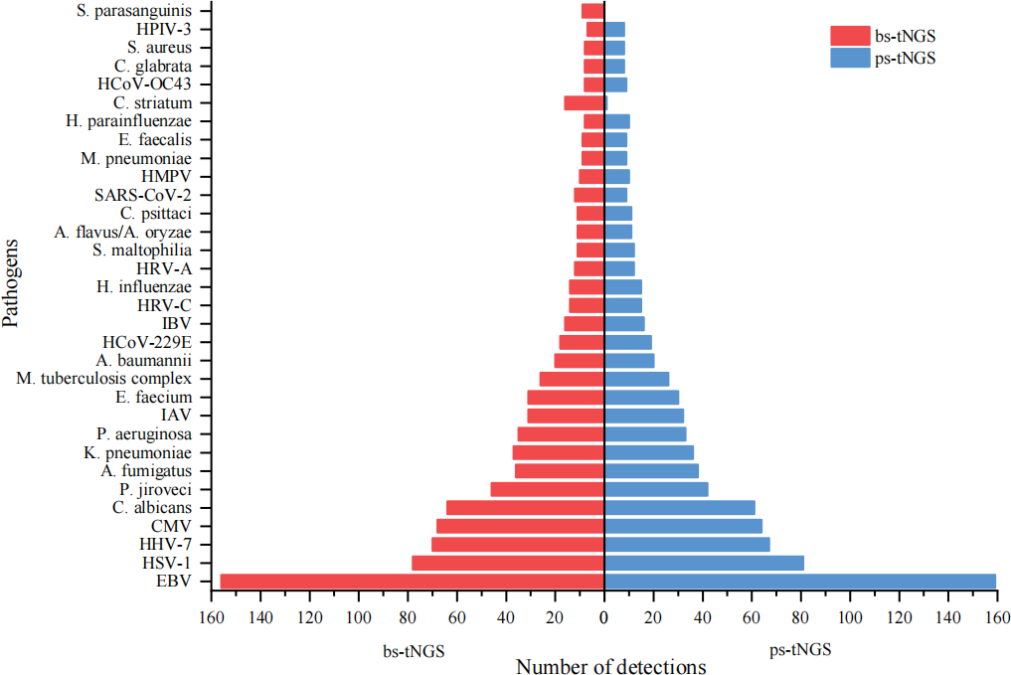
The 30 most frequently detected pathogens by bs-tNGS and ps-tNGS. Bar graph showing the number of detections of the top 30 pathogens reported by the two methods.

We analyzed the prevalence of reported pathogens and discovered that the top 50 accounted for 90.04% (1,004 out of 1,115) and 96.41% (967 out of 1,003) of all detections by bs-tNGS and ps-tNGS, respectively (Fig. 2A and B). A similar trend was also observed in the top 30 pathogens, which showed a significant overlap (93.33%, 28 out of 30) between the two methods (Fig. 3). Notably, the most frequently detected pathogens were almost equivalent, including EBV, HSV-1, human beta herpesvirus 7, CMV, *C. albicans*, *Pneumocystis jirovecii*, *Aspergillus fumigatus*, *K. pneumoniae*, *Pseudomonas aeruginosa*, *M. tuberculosis* complex, influenza A virus, *Enterococcus faecium*, *Acinetobacter baumannii*, and human coronavirus 229E.

### Comparison of diagnostic accuracy

**Fig 4:**
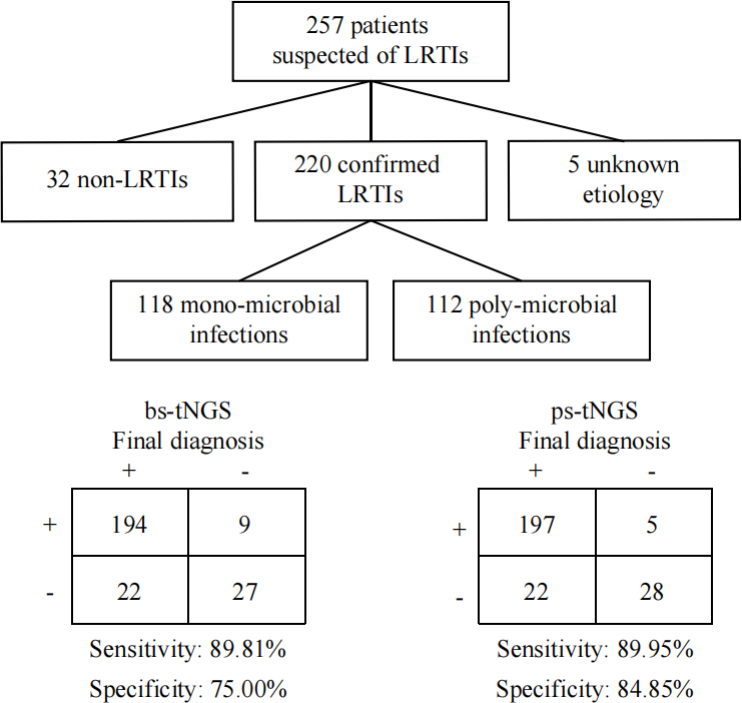
The diagnostic performances of bs-tNGS and ps-tNGS. A total of 257 patients with suspected LRTIs were enrolled in this study, and the sensitivities and specificities were compared between bs-tNGS and ps-tNGS, respectively.

We then evaluated whether the two assays performed differently in the etiological diagnosis of LRTIs. To that end, a multidisciplinary panel of three experts (a physician, a clinical microbiologist, and an infectious disease specialist) reviewed the electronic health records, including the patients’ symptoms, underlying diseases, imaging results, microbiological results, and antibiotic usage. The adjudications were made by thoroughly discussing all available clinical and diagnostic information, including the results from CMTs, bs-tNGS, and ps-tNGS. As shown in Fig. 4, 32 patients were diagnosed with non-infectious diseases (non-LRTIs), including chronic obstructive pulmonary disease, lung cancer, radiation pneumonitis, and interstitial lung disease. A total of 220 patients were diagnosed with LRTIs, while the etiology of five patients could not be determined despite comprehensive record review and discussions. In 220 cases of LRTIs, 118 (53.64%) were mono-microbial infections and 102 (46.36%) were poly-microbial infections (bacteria + fungi, bacteria + viruses, fungi + viruses, and bacteria + fungi + viruses). Using the final diagnosis as a reference, bs-tNGS had 9 false positives, 194 true positives, 27 true negatives, and 22 false negatives (missed one or more pathogens that had been identified by CMTs), resulting in a diagnostic sensitivity of 89.81% (95% confidence interval [CI]: 84.99%–93.51%) and specificity of 75.00% (95% CI: 57.80%–87.88%). On the other hand, the ps-tNGS assay had 5 false positives, 197 true positives, 28 true negatives, and 22 false negatives, resulting in a diagnostic sensitivity of 89.95% (95% CI: 85.19%–93.60%) and specificity of 84.85% (95% CI: 68.10%–94.89%) (Table 2; Table S2).

## DISCUSSION

The shotgun metagenomic sequencing (mNGS) has been used to detect pathogens from BALF and sputum samples for diagnosing LRTIs (26–28). In contrast, tNGS approaches were primarily used in 16S rRNA or ITS sequencing in microbiome studies (29). In 2020, pathogen-specific enrichment was achieved in NGS assays by multiplex PCR to amplify 36 pathogens and 49 antibiotic resistance markers from sputum for diagnosing acute respiratory tract infections and predicting drug resistance (30). In 2022, an in-house tNGS workflow was compared with mNGS to assess their performances in diagnosing respiratory infections. Using a composite clinical standard consisting of CMT, chart review, and orthogonal testing, the overall accuracy of tNGS was determined to be 65.6%, while the metagenomic approach showed a similar accuracy of 67.1% (31). More recently, one study has evaluated the clinical performances of two tNGS assays in a prospective cohort of 251 patients diagnosed with LRTIs. One used multiplex PCR (multiplex polymerase chain reaction-based targeted next-generation sequencing [mp-tNGS]), while the other used hybrid capture (hybrid capture-based targeted next-generation sequencing [hc-tNGS]). It was found that mp-tNGS and hc-tNGS showed 84.3% and 89.5% accuracy (88.5% for mNGS) in detecting causal pathogens from BALF samples, respectively. *P. jirovecii* was detected by both tNGS workflows but missed by mNGS in seven samples, while mNGS was better than tNGS in detecting filamentous fungi (6).

In our study, both tNGS assays were similar in terms of the positive rate and diagnostic performances in respiratory samples. The most commonly detected pathogens were highly similar between the two assays, suggesting that a small subset of pathogens was frequently occurring among all LRTI cases. Notably, the human herpesviruses (EBV, HSV-1, HSV-7, and CMV) were highly prevalent in the respiratory tract, accounting for 33.36% and 36.99% of all reported microorganisms by bs-tNGS and ps-tNGS, respectively. These findings need to be interpreted with caution since latent infections of herpesviruses are common, and they may become active infections in individuals with weakened or compromised immune systems (32). Upon detection of these viruses, clinicians may consider antiviral treatment for the patients if they are clearly immunocompromised, especially if they have undergone organ or bone marrow transplant.

Ten microbial species were reported by bs-tNGS that were not covered by ps-tNGS since they were of low virulence and often considered to be colonizers of the respiratory tract, such as *Prevotella* spp. (33). This could be both a disadvantage and advantage at the same time since these microorganisms generally cause no signs or symptoms in immunocompetent individuals but may cause LRTIs in immunocompromised patients. Reporting of these microorganisms may potentially lead to overuse of antibiotics. Due to the broad scope of detection, interpretation of results remains to be one of the biggest challenges of mNGS (34). Narrowing down the detection scope to obligate pathogens or species that are highly likely to be causative pathogens may not only simplify the interpretation of results but also promote the reasonable use of antibiotics. Therefore, ps-tNGS was designed to mainly cover common respiratory pathogens and some less prevalent but clinically actionable pathogens (such as *Rickettsia* spp. and *Chlamydia* spp.), resulting in a higher specificity than bs-tNGS. In addition, the assay cost of ps-tNGS is significantly lower than that of bs-tNGS due to a huge difference in the number of detectable pathogens between these two methods.

On the other hand, due to the differences in primer design, bs-tNGS had a broader coverage of pathogens and was able to identify the subtype/subspecies more efficiently than ps-tNGS. For instance, in one case, bs-tNGS detected H3N2, whereas ps-tNGS reported only influenza A virus (case 31, Table S2). Though it may not affect the diagnostic decision and prescription of antiviral medications, bs-tNGS might be better suited for the purpose of pathogen surveillance, especially for endemic viruses with multiple subtypes. Similarly, in cases 97 and 200, bs-tNGS detected *Rhizomucor miehei* and HPIV-4, respectively, whereas ps-tNGS reported only *Rhizomucor* and *Orthorubulavirus*.

There are a number of limitations in this study: (i) this was a single-center study design; (ii) this study focused only on inpatients; (iii) the patients were selected based on imaging results and clinical presentations that might be biased; and (iv) the final diagnosis was made according to multidisciplinary team discussion, and determining the causative pathogens was challenging, especially in polymicrobial infections.

In summary, both bs-tNGS and ps-tNGS assays took advantage of multiplex PCR for pathogen enrichment and exhibited similar sensitivity and accuracy for identification of respiratory pathogens, but ps-tNGS showed higher specificity than bs-tNGS. Therefore, tNGS, with specific enrichment of clinically relevant respiratory pathogens, has the potential to serve as a new and efficient approach for the diagnosis of LRTIs. Future prospective validation studies are needed to further explore the clinical utility of these tNGS assays in respiratory and other infectious diseases. Moreover, automation of experimental procedures may be developed to optimize the workload and reduce turnaround time (35).

## SUPPLEMENTAL MATERIAL

Tables S1 and S2 spectrum.00922-25-s0001.xlsx Table S1. The pathogens reported by two tNGS workflows. Table S2. Clinical records and testing results of all enrolled patients.

ASM does not own the copyrights to Supplemental Material that may be linked to, or accessed through, an article. The authors have granted ASM a non-exclusive, world-wide license to publish the Supplemental Material files. Please contact the corresponding author directly for reuse.
